# Pyogenic Liver Abscess and Sepsis Caused by Streptococcus constellatus in the Immunocompetent Host

**DOI:** 10.7759/cureus.9802

**Published:** 2020-08-17

**Authors:** Muhammad Z Khan, Danial Tahir, Asim Kichloo, Nicholas Haddad, Abdul Hanan

**Affiliations:** 1 Internal Medicine, Central Michigan University College of Medicine, Saginaw, USA; 2 Pediatrics, Ayub Medical College, Abbottabad, PAK; 3 Internal Medicine, Central Michigan University, Saginaw, USA; 4 Infectious Disease/Internal Medicine, Central Michigan University College of Medicine, Saginaw, USA; 5 Infectious Disease, Wayne State University, Detroit, USA

**Keywords:** liver abscess, pyogenic, cultures, drainage, sepsis, streptococcus constellatus, pigtail catheter

## Abstract

Streptococcus constellatus is a member of Streptococcus milleri group which is a subgroup of Viridans streptococci, first described by Guthof in 1956 after being isolated from dental abscesses. S. constellatus, a gram positive, non-sporing, non-motile, catalase negative cocci, is the normal flora of the oropharyngeal, gastrointestinal and urogenital tract. It is not a commonly encountered pathogen but has a propensity to form abscesses and cause bacteremia in the immunocompromised patient. Here, we report a 78-year-old man with sepsis due to Streptococcus constellatus liver abscess. The patient had a history of hypertension, stroke, benign prostatic hyperplasia, vascular dementia and myocardial infarction status post coronary artery bypass grafting. There has been no particular link between any of these conditions to S. constellatus. However, immunocompromised status predisposes to fulminant infection and formation of abscesses. The patient was febrile with a temperature of 99.1°F, blood pressure of 143/73 mmHg and the heart rate (HR) of 98. Labs revealed a leukocytosis of 16.90 K/uL, hemoglobin 11.8 g/dL, hematocrit 35.8%, total bilirubin 1.7 mg/dL, direct bilirubin 1.0 mg/dL, aspartate aminotransferase (AST) 44 IU/L, alanine aminotransferase (ALT) 28 IU/L, alkaline phosphatase (ALKP) 176 IU/L and lactate dehydrogenase (LDH) was 290 IU/L. He was started on intravenous Maxipime and Unasyn which was switched to Rocephin and Clindamycin based on the Infectious disease recommendations. Metronidazole was also started and the serologies were sent for Entamoeba histolytica. Computerized tomography (CT) scan showed an abscess in the right lobe of the liver which was finally drained using an interventional radiology (IR)-guided approach. The cultures from the fluid and blood yielded S. constellatus and thus Metronidazole was discontinued. The patient improved after a few days and the drainage catheter was pulled out and the patient discharged in stable condition.

## Introduction

Liver abscess is a potential life-threatening disease that must be treated with drainage and bacteriological studies performed as soon as possible which allows isolation of causative agents and specific antibiotic therapy [1]. Liver abscess is most frequently associated with biliary tract disorders, however, it may also develop from hematogenous dissemination of organisms in association with systemic bacteremia such as endocarditis and pyelonephritis. The first case of Streptococcus constellatus hepatic abscess was reported in 1975. Since then they have been identified as one of the important pathogens of liver abscess. Streptococcus constellatus, anginosus and intermedius belong to the milleri subgroup of organisms [2,3]. They reside as part of the normal flora of the respiratory, gastrointestinal and urogenital tracts [4,5]. Despite this, their potential to form abscesses has been reported which also depends on factors related to the human host.

## Case presentation

**Figure 1:**
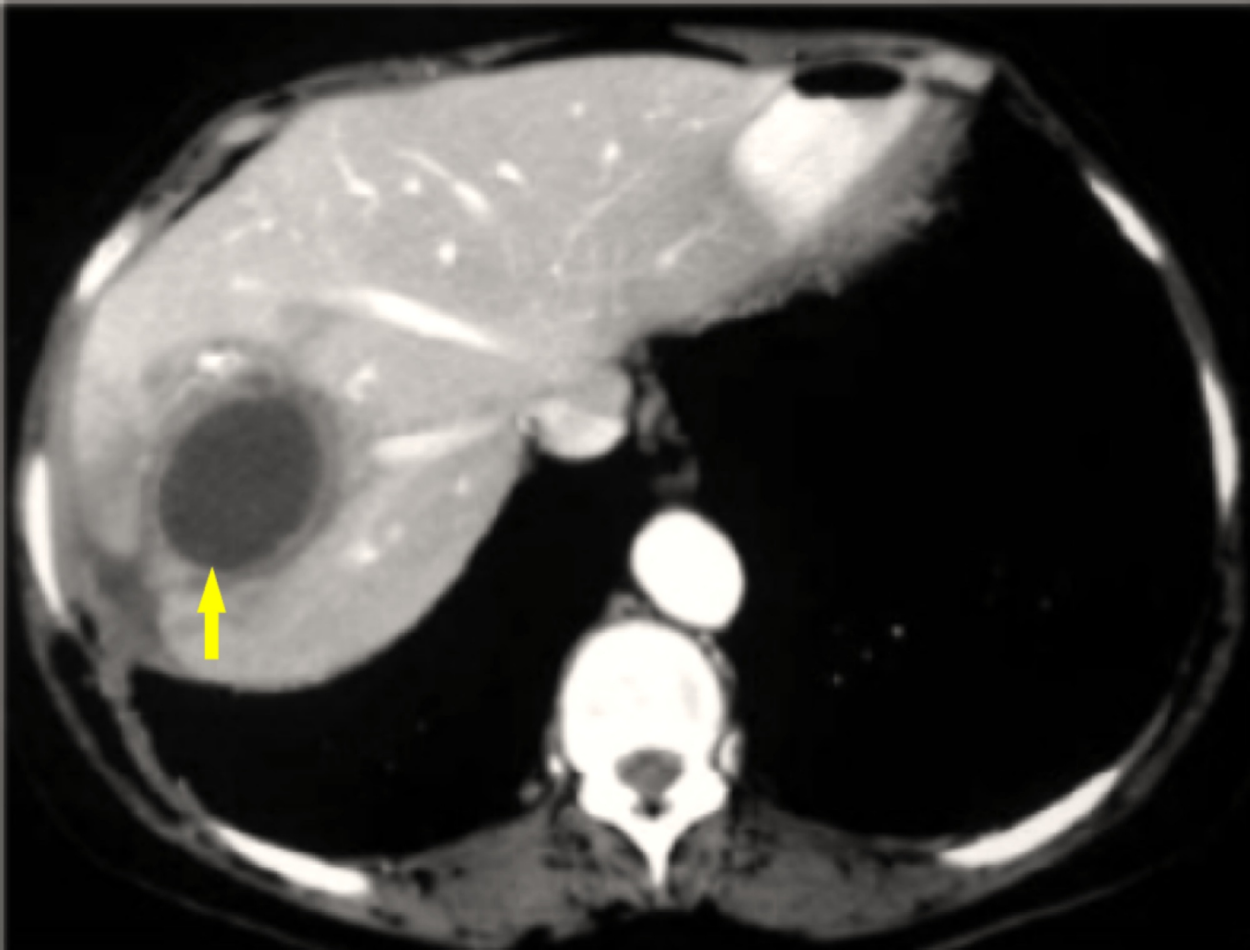
CT scan with contrast shows fluid collection in the right lobe of the liver surrounded by the rim of hypoattenuation (yellow arrow).

A 78-year-old African American man with a past medical history of hypertension, stroke, benign prostatic hyperplasia, vascular dementia and myocardial infarction status post coronary artery bypass grafting (CABG) was brought to the emergency room by his family with the chief complaint of chills and fever for one day. He denied any shortness of breath, palpitations, cough, headache, diarrhea and constipation. The patient immigrated to the United States one month ago from Haiti. On examination, the patient’s temperature was 99.1°F, heart rate (HR) 98 and blood pressure (BP) of 143/73 mmHg. He complained of right upper quadrant pain upon light palpation. Laboratory findings on admission were as follows: white blood cell (WBC) 16.90 K/L (Normal range = 4.5-11 K/L), hemoglobin 11.8 g/dL (Normal range = 13.5-17.5 g/dL), hematocrit 35.8% (Normal range = 41-50%), total bilirubin 1.7 mg/dL (Normal range = 0.1-1.2 mg/dL), direct bilirubin 1.0 mg/dL (Normal range = less than 3 mg/dL), aspartate aminotransferase (AST) 44 IU/L (Normal range = 6-34 IU/L), alanine aminotransferase (ALT) 28 IU/L (Normal range = 29-33 IU/L), alkaline phosphatase (ALKP) 176 IU/L (Normal range = 37-116 IU/L) and lactate dehydrogenase (LDH) 290 IU/L (Normal range = 105-333 IU/L). The patient was admitted to the floor and started on 1 g IV Maxipime Q12 hr, 3 g IV Unasyn Q8 hr and IV normal saline 63 cc/hr for sepsis, pending blood and urine cultures. Twelve hours later the patient’s temperature increased to 104.1°F, blood pressure of 148/72 and WBC 24 K/uL and infectious disease (ID) was called for consultation. On day 3, the patient’s WBC continued to be elevated at 23.8 K/uL. Blood cultures came back positive for Streptococcus constellatus. Following this current treatment, the patient’s temperature stabilized to 98.4°F and WBC decreased to 19.8 K/uL, however, the patient’s AST and ALT kept trending up which led to an abdominal CT scan which showed irregular fluid changes in the hepatic parenchyma (Figure 1).

E. histolytica ELSIA serology was ordered. Interventional radiology was consulted for abscess drainage and Metronidazole 500 mg Q8 hr was added to the treatment regimen. On day 5 after admission the intrahepatic abscess was drained under CT guidance and a pigtail catheter was placed. At that time, infectious disease suggested starting clindamycin 900 mg IV Q8 hr and Rocephin 2 g IV Q24 hr. On day 7, the liver abscess culture came back positive for Streptococcus constellatus and negative for malignant cells, therefore, IV metronidazole was discontinued. The blood cultures revealed the growth of Streptococcus constellatus. On day 8, CT of the abdomen without contrast was done which showed the pigtail catheter in the left lobe of the liver, status post liver abscess drainage. There was marked decrease in the amount of low-attenuation inflammatory change in the area. The remainder of the liver appeared normal; hence the catheter was released and removed. On day 16, the patient was discharged with stable vitals of temperature of 97.8°F, pulse 66, blood pressure 100/59 and WBC 8.01 K/uL.

## Discussion

Streptococcus constellatus belongs to the Streptococcus milleri group which also includes S. anginosus and intermedius [6]. They reside as commensals in the oropharynx and the gastrointestinal tract. Sometimes, due to mechanisms partly unexplained, these organisms become pathogenic and acquire the potential to form abscesses at sites which include but are not limited to the urogenital tract, skin, abdominal and oral cavities. Direct spread to a contiguous site or hematogenous dissemination is reported. Streptococcus constellatus being a member of the milleri subgroup, has the predisposition to form abscesses but as stated earlier, the exact reason is not clear [7]. Possibilities include the organism’s polysaccharide capsule and synergistic activity with anaerobes. The occurrence of bacteremia is related to host factors which include diabetes mellitus, chronic kidney disease, hepatobiliary disease, neoplasia and treatment with chemotherapeutic drugs which compromise the immune response [8,9]. Often times a suppurative focus of infection can be located in the gastrointestinal tract, lungs (related to aspiration pneumonia) or urogenital tracts. Disruption of the mucosal barrier facilitates as a portal of entry. Penicillins and cephalosporins are effective treatment options for Streptococcus constellatus. Variable susceptibility to tetracycline, clindamycin, and erythromycin has been reported [10]. However, the definitive treatment for pyogenic abscesses remains aspiration and drainage with tailoring of the antibiotics according to the susceptibility results. Our case illustrates the unusual presentation of severe sepsis from Streptococcus constellatus bacteremia. The knowledge of the different clinical associations of S. constellatus can aid in the management and the search for an associated infection or occult abscess [11].

## Conclusions

Streptococcus constellatus has been an important cause of pyogenic abscesses. Immunocompromised status puts the patient at risk of dissemination and formation of remote foci of infection. Our case highlights the importance of having a low suspicion for a liver abscess in certain conditions in the immunocompetent patient so that appropriate therapy can be instituted and complications avoided.
